# ASO Author Reflections: Clinical Staging of Localized Colon Cancer: Room for Improvement

**DOI:** 10.1245/s10434-024-15956-9

**Published:** 2024-07-29

**Authors:** Sameh Hany Emile, Steven D. Wexner

**Affiliations:** 1 Ellen Leifer Shulman and Steven Shulman Digestive Disease Center, Cleveland Clinic Florida, Weston, FL USA; 2 Colorectal Surgery Unit, General Surgery Department, Mansoura University Hospitals, Mansoura, Egypt

Preoperative clinical assessment of colon cancer is imperative to assess the TNM stage of the disease and help guide management. Clinical assessment is undertaken using imaging modalities, including computed tomography (CT), magnetic resonance imaging (MRI), and positron emission tomography (PET)/CT. CT scanning is perhaps the most widely used modality for clinical assessment. Despite having good diagnostic accuracy in identifying locally advanced T3-T4 colon cancers, the accuracy of a CT scan in the detection of nodal disease is limited, with a sensitivity and specificity of 62% and 70%, respectively.^1^

The present study^2^ found that clinical assessment of the T and N stages of colon cancer had only moderate agreement with the final pathologic stages. The agreement was more pronounced for the T stage in left colon cancers. While clinical assessment was highly specific for the T and N stages, it had a lower sensitivity of 64.3–77.2% for T stage and 41.6–54.5% for N stage, lower than what has been previously reported.^1^ One important observation was that the accuracy of clinical assessment in the detection of advanced TN stages was higher than that for earlier stages, as 90.1% of stage III patients were correctly staged compared with 60.4% of stage I patients. The poor accuracy of clinical assessment of nodal disease may be attributable to patient and tumor characteristics. A recent study^3^ found patients <65 years of age, patients with left-sided and high-grade cancers and elevated carcinoembryonic antigen (CEA) were more likely to have their nodal status clinically understaged.

The findings of the present study have important implications, as clinical assessment is more accurate in patients with advanced stage and thus neoadjuvant chemotherapy may be indicated in these patients. Conversely, approximately 25% of patients with advanced colon cancer would be clinically understaged, thus patients identified in imaging studies to have early-stage disease may need to be thoroughly assessed with other imaging modalities before surgery.
